# Analysis of reported incidence of chronic hepatitis B in Chaoyang District, Beijing during 2005–2022 with age-period-cohort model

**DOI:** 10.3389/fpubh.2025.1486475

**Published:** 2025-03-13

**Authors:** Qian Li, Xuerou Zhao, Han Meng, Qianlan Zhang, Xuan Liu, Xiaohong Jiang, Xiao Qi

**Affiliations:** ^1^Department of Epidemiology and Endemic Disease Control, Chaoyang District Center for Disease Prevention and Control, Beijing, China; ^2^Department of Social Medicine and Health Education, School of Public Health, Peking University, Beijing, China

**Keywords:** chronic hepatitis B (CHB), age-period-cohort model, Intrinsic Estimator (IE), birth cohort, incidence of CHB

## Abstract

**Objective:**

The study aimed to describe the trend of chronic hepatitis B among the population in Chaoyang District from 2005 to 2022 and explore the effects of age, period, and cohort factors on the incidence risk.

**Methods:**

Incidence rates of chronic hepatitis B were collected from the national infectious disease reporting and information management system. Descriptive epidemiological methods were employed to characterize the incidence of chronic hepatitis B from 2005 to 2022. Trends in chronic hepatitis B were analyzed using an age-period-cohort model. The effects of age, period, and cohort on chronic hepatitis B incidence were estimated using the Intrinsic Estimator operator. Model goodness of fit was assessed by introducing variance, AIC, and BIC, comparing the established model with conventional two-factor models.

**Results:**

From 2005 to 2022, the reported incidence of chronic hepatitis B in Chaoyang District showed a gradual decrease followed by stabilization, declining from 49.58/100,000 to 8.58/100,000 overall, from 63.36/100,000 to 11.69/100,000 in males, and from 35.15/100,000 to 5.55/100,000 in females. The age effect coefficient for males with chronic hepatitis B increased initially and then decreased with age, whereas for females, it decreased initially and then increased with age. The period effect on chronic hepatitis B incidence risk in both genders initially decreased and then increased over time. The cohort effect coefficient for males exhibited a pattern of decrease, increase, and then decrease, whereas for females, it generally increased and then decreased. The APC model constructed with the intrinsic estimator demonstrated the best goodness of fit, as indicated by lower variance, AIC, and BIC compared to conventional two-factor models.

**Conclusion:**

The reported incidence of chronic hepatitis B in Chaoyang District from 2005 to 2022 declined and stabilizing after 2013. We found distinct age, period, and cohort effects on the incidence, with higher risks observed in males aged 25–29 and 75–79, and females born in 1966–1970. These findings highlight the importance of targeted surveillance, expanded screening, and improved diagnosis and treatment rates to reduce the long-term chronic hepatitis B burden.

## 1 Introduction

Hepatitis B is caused by the Hepatitis B virus (HBV), an infectious disease primarily transmitted through blood, leading to liver inflammation and necrosis (1). By 2022, approximately 254 million people worldwide were infected with chronic hepatitis B, with 1.23 million new infections and 1.1 million deaths attributed to HBV annually. China alone accounts for about 79.74 million chronic hepatitis B cases, constituting 31.5% of the global total. In 2022, deaths due to HBV infection in China are projected to exceed 450,000, representing 41.2% of global HBV-related deaths (2). Chaoyang District in Beijing serves as a model area for comprehensive viral hepatitis prevention and treatment, implementing a range of measures. The HBsAg positivity rate in Beijing decreased from 3.02% in 2006 to 2.68% in 2015 specifically in Chaoyang District (3). Currently, there is a lack of literature exploring the incidence patterns and changing trends of hepatitis B in Chaoyang District. The World Health Organization (WHO) has set a goal to eliminate hepatitis B by 2030, aiming for a 90% reduction in new infections and a 65% reduction in mortality rates compared to 2015 levels (4). There is an urgent need to develop a comprehensive prevention and control plan tailored to residents of Chaoyang District and to clarify the epidemiological patterns of chronic hepatitis B in the area.

The trend of disease changes is often explained through the lenses of age, period, cohort, and random variation. Traditional statistical description methods and research designs struggle to eliminate or control the interactions between these factors, thereby failing to accurately reflect their real impact on incidence rates (5). The Age-Period-Cohort (APC) model addresses these limitations by quantifying the effects of age, period, and birth cohort (6). Widely applied in analyzing time trends in the onset or mortality of chronic diseases, the APC model has expanded into the realm of infectious diseases.

The APC model is a standard statistical analysis tool in demography, sociology, and epidemiology. In recent years, it has been extensively utilized to analyze trends in incidence rates and mortality for chronic non-communicable diseases, forecasting disease burden changes over future years or decades. By overcoming the deficiencies of traditional methods that cannot differentiate age, period, and cohort effects, the APC model, based on the Poisson distribution, estimates disease risk under varying age, period, and birth cohort conditions.

In this study, age denotes the age of onset for cases, with the age effect representing how the incidence rate changes with age. Period refers to the year of occurrence for cases, and period effect signifies variations in disease rates influenced by human factors such as advancements in disease diagnosis technology, screening practices, early detection methods, changes in disease definition and registration criteria during specific periods, and other external factors impacting disease rates. Birth cohort indicates the year of birth for cases, and cohort effect denotes the cumulative impact of all exposure factors such as socio-economic environment, historical events, and lifestyle choices since birth on disease rates (7).

The classic APC model, based on the Poisson distribution and employing a logarithmic linear regression framework, can effectively illustrate the risk and trend of disease onset or mortality within a given population while simultaneously adjusting for factors such as age, period, and cohort. The general form of its equation is:


$$\begin{array}{l} \ln\left(\frac{y_{ij}}{n_{ij}}\right)=\mu +\alpha_{i}+\beta_{j}+\gamma_{k} \end{array}$$

In the equation, *y*_*ij*_ represents the number of cases or deaths, assuming that it obeys the independent Poisson distribution; *n*_*ij*_ represents the number of population; μ represents the disease risk reference level of age, period, and cohort parameters, and usually the first level of the parameter is a reference to other levels; α_*i*_ represents the age effect, and *i* is age group (*i* = 1, 2, 3...); β_*j*_ represents the period effect, and *j* is the specific year (*j* = 1, 2, 3...); γ_*k*_represents the effect of birth cohort *k*, which refers to the birth cohort effect related to the age of layer i and the period of layer *j*; variable *k* represents the independent effect of birth cohort, *k* = *j*-*I* + *n*_*a*_; *n*_*a*_ represents the number of age groups (6).

Since there exists a linear relationship among age, period, and birth cohort—that is, birth cohort equals the difference between period and age—the APC model described earlier encounters a challenge known as the “unidentifiability” problem, where no unique set of parameter solutions exists theoretically. Various methods have been developed to address this issue, including local restriction methods, two-factor models, characteristic variable methods, non-linear approaches, penalty function methods, autoregressive techniques, individual observation methods, estimation function approaches, non-parametric causal models, and non-parametric smoothing spline functions (6). However, each method carries its own limitations. Fu and Land introduced the Intrinsic Estimator (IE) algorithm, also known as the endogenous factor method, which overcomes these challenges by satisfying model estimability conditions and ensuring the identification of a unique solution without requiring restrictive assumptions (8). This method has gained popularity for its robustness and applicability across various contexts.

This study utilized data on chronic hepatitis B cases reported in Chaoyang District, Beijing from 2005 to 2022, obtained from the Infectious Disease Reporting Information Management System. It applied the APC model alongside the Intrinsic Estimator (IE) to investigate the influence of age, period, and birth cohort factors on the incidence trends of chronic hepatitis B.

## 2 Data and method

### 2.1 Data

The reported cases of chronic hepatitis B were collected from the Infectious Disease Monitoring Information System of the China Information System for Disease Control and Prevention. Surveillance data included chronic hepatitis B cases residing in Chaoyang District for more than 6 months, spanning from January 1, 2005, to December 31, 2022. Diagnosis criteria followed the HBV standards set by the Chinese Ministry of Health, based on clinical symptoms or laboratory test results (9), and all cases were reported online within 24 h of diagnosis. Population data for Chaoyang District were sourced from the National Infectious Disease Reporting Information Management System, while population data for each sub-district within Chaoyang District were derived from basic demographic information within the Beijing Immunization Planning Information System.

The chronic hepatitis diagnosis criteria list was as follows.

1. Acute HBV infection for more than 6 months and still HBsAg positive or found HBsAg positive for more than 6 months.
2. HBsAg positive duration is unknown, anti-HBc IgM negative.
3. Signs of patients with chronic liver diseases such as liver face, liver palms, spider nevus and hepatosplenomegaly.
4. Repeated or sustained elevation of serum ALT may be associated with a decrease in plasma albumin and/or an increase in globulin or bilirubin.
5. Liver pathology has the characteristics of chronic viral hepatitis.
6. Serum HBeAg was positive or HBV DNA could be detected, and other causes of elevated ALT were excluded.

The diagnosis of chronic hepatitis B can be made if the case meets the above combination (146, 156, 246, 256).

### 2.2 Method of analysis

Descriptive methods were employed to analyze the three-dimensional distribution of reported chronic hepatitis B cases in Chaoyang District from 2005 to 2022. Cases aged 20–84 years were included in the APC trend and model analysis. The APC model combined with the Intrinsic Estimator (IE) method was utilized for data analysis. The APC model encompassed three target variables: age, period, and birth cohort (10). Age effect quantifies differences in disease incidence and prevalence across various age groups. Period effect captures changes in disease incidence and prevalence due to economic shifts, advancements in diagnostic techniques and screening practices, and alterations in social and demographic factors. Cohort effect describes changes in disease incidence and prevalence resulting from exposure to different historical events among individuals born at different times (6, 11, 12).

### 2.3 Statistical analysis

Excel 2016 was utilized to analyze the three-dimensional distribution of reported chronic hepatitis B incidence. The APC model was constructed using the apc_ie software package and IE algorithm in STATA 17.0 (MP). The effect coefficient was calculated with a test level α = 0.05. Additionally, the traditional two-factor model was constructed using the SAS 9.2 genmod program. Model fitting was evaluated using Deviance, Akaike Information Criterion (AIC), and Bayesian Information Criterion (BIC).

## 3 Results

### 3.1 Incidence of chronic hepatitis B reported

**Figure 1:**
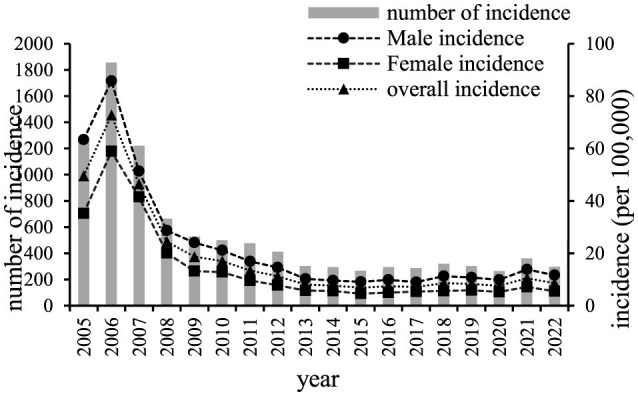
Reported incidence of chronic hepatitis B in Chaoyang District, Beijing, from 2005 to 2022.

**Figure 2:**
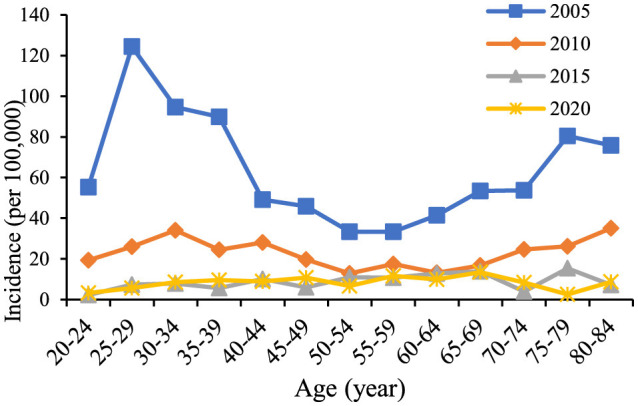
Trends in the incidence of chronic hepatitis B among residents aged 20–84 years in Chaoyang District from 2005 to 2020.

During 2005 to 2022, 9,902 cases of chronic hepatitis B were reported in Chaoyang District, accounting for 88.20% of all reported hepatitis B cases. The annual reported incidence of chronic hepatitis B was 16.40 per 100,000. The total reported incidence of chronic hepatitis B exhibited a declining trend, decreasing from 49.58 per 100,000 in 2005 to 8.58 per 100,000 in 2022 (Figure 1). Of the reported cases, 6,250 were male (annual incidence 22.8 per 100,000) and 3,652 were female (14.16 per 100,000), resulting in sex ratios ranging from 1.24 to 2.11 each year. The highest number of reported cases occurred in the 25–34 age group, totaling 2,588 cases. The highest incidence rates were observed in the age group over 85 years old (27.04 per 100,000), followed by the 40–44 age group (22.44 per 100,000) (Figure 2).

### 3.2 The analysis of APC trend

**Figure 3:**
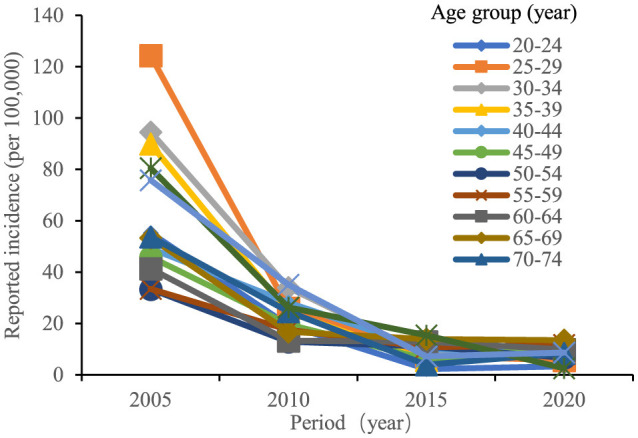
Age-specific incidence trends of chronic hepatitis B among residents aged 20–84 years in Chaoyang District from 2005 to 2020.

**Figure 4:**
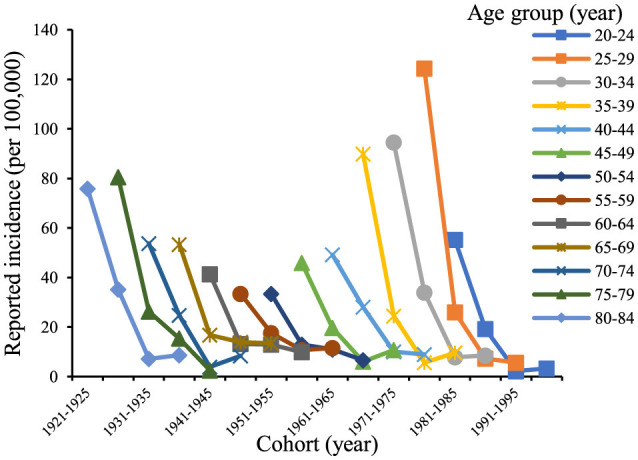
Trend of incidence of chronic hepatitis B in birth cohorts of residents aged 20–84 years in Chaoyang District from 2005 to 2020.

The age trends in reported incidence of chronic hepatitis B in the total population aged 20–84 years in Chaoyang District over time (Figure 2) revealed peak incidence rates in the 25–34 and 75–84 age groups in 2005 and 2010. Across different age groups, there was a consistent decreasing trend in reported incidence over time. Figure 3 illustrates decreasing reported incidence trends over time across various age cohorts, with slightly higher rates observed in 2020 compared to 2015 among the 55–59, 70–74, and 80–84 age groups. Trends in the birth cohort (Figure 4) depict declining incidence rates across all age groups initially, with a slight increase observed in the 20–24, 30–34, 35–39, 45–49, 55–59, 70–74, and 80–84 age groups over the birth cohort.

**Figure 5:**
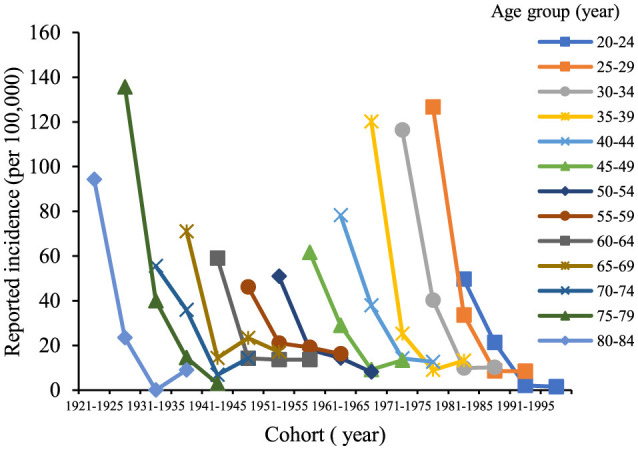
Trend of incidence of chronic hepatitis B in male birth cohorts aged 20–84 years in Chaoyang District from 2005 to 2020.

**Figure 6:**
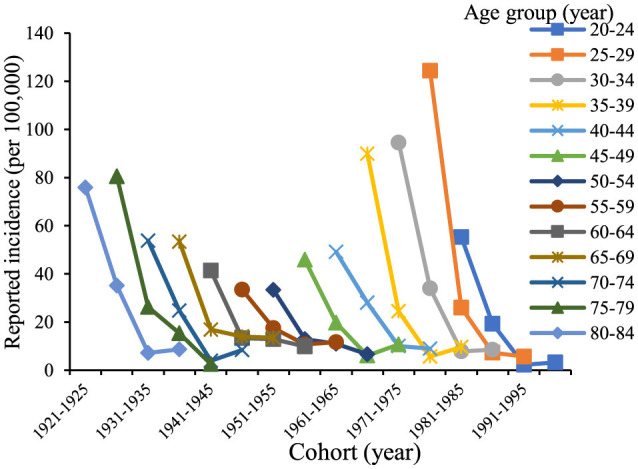
Incidence trend of chronic hepatitis B in female birth cohorts aged 20–84 years in Chaoyang District from 2005 to 2020.

In Chaoyang District, the incidence of chronic hepatitis B by gender from ages 20 to 84 indicated that for males (Figure 5), the incidence rates of each age group initially decreased and then stabilized as the birth cohort progressed. Specifically, the age groups 50–54, 55–59, and 75–79 years exhibited a continuous downward trend, while the age groups 35–39, 45–49, 70–74, and 80–84 years showed a decline followed by a slight increase. For females, the reported incidence rates of each age group showed a declining trend with birth progression. Overall, the reported incidence rates across all age groups in the population demonstrated a decreasing trend over time, although the rates for the 20–24, 30–39, 45–49, 55–59, 65–69, and 70–74 age groups in 2020 showed a slight increase compared to those in 2015 (Figure 6).

### 3.3 The analysis of APC model

Comparison of age-period-cohort models, including the traditional two-factor models (age-period, age-cohort, and period-cohort) fit for the reported incidence data of chronic hepatitis B in the whole population, men, and women showed the best fit of the APC model (Variance = 53.20, AIC = 7.34, BIC = −33.72) (Table 1).

**Table 1:** Fitting test results of the age-period-cohort model for reported incidence of chronic hepatitis B in Chaoyang District, Beijing.

**Model**	**Totally**			**Males**			**Females**		
	**Variance**	**AIC**	**BIC**	**Variance**	**AIC**	**BIC**	**Variance**	**AIC**	**BIC**
APC	53.20	7.34	−33.72	46.17	6.69	−40.76	45.55	6.14	−41.37
AP	162.85	456.41	487.62	116.45	390.07	421.29	116.74	362.60	393.82
AC	119.17	436.72	491.36	126.95	424.57	479.20	89.57	359.42	414.06
PC	120.60	420.15	457.22	96.55	376.16	413.24	106.40	358.26	395.33

**Figure 7:**
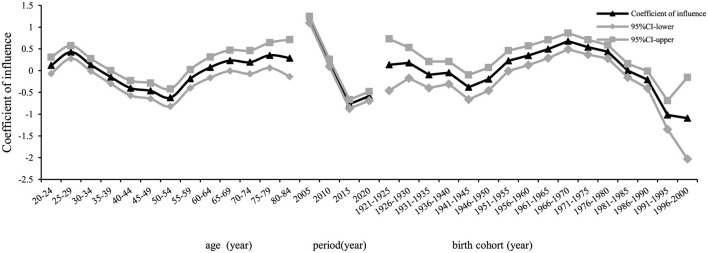
Age, period, and birth cohort effect coefficients of reported incidence of chronic hepatitis B among residents aged 20–84 years in Chaoyang District.

**Figure 8:**
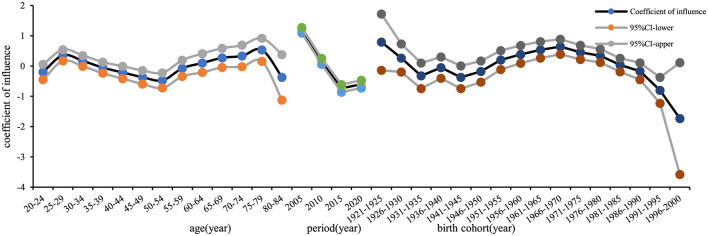
Age, period, and birth cohort effect coefficients of reported incidence of chronic hepatitis B among males aged 20–84 years in Chaoyang District.

**Figure 9:**
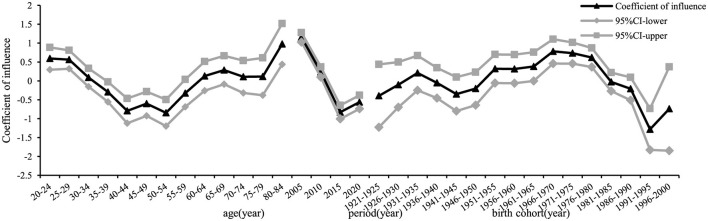
Age, period, and birth cohort effect coefficients of reported incidence of chronic hepatitis B among females aged 20–84 years in Chaoyang District.

The age effect coefficient on the risk of developing chronic hepatitis B in the whole population of Chaoyang District increased from 0.12 in the age group 20–24 to 0.42 in the age group 25–29, then decreased to −0.62 in the age group 50–54, with a subsequent increase to 0.35 in the age group 75–79, followed by a slight decrease. Relative to the age group 20–24, the relative risk (RR) for the age group 25–29 was 1.36 (Table 2). The period effect on incidence risk declined initially and then increased over time, with an *RR* for the incidence risk effect coefficient of 0.17 in 2020, using 2005 as the reference year. The contribution of the birth cohort to the incidence of chronic hepatitis B increased initially and then declined, with an *RR* of 1.72 for the 1966–1970 birth cohort, using the years 1921–1925 as the reference (Figure 7). The trends of age, period, and cohort effects for chronic hepatitis B in the male and female populations mirrored those of the whole population. Higher values were observed in the male population in the 75–79 age group (*RR* = 2.08, effect coefficient = 0.53, *95% CI*: 0.15–0.91) and the 25–29 age group (*RR* = 1.74, effect coefficient = 0.35, *95% CI*: 0.17–0.54) (Figure 8). In the female population, the 1966–1970 birth cohort had the highest effect coefficient (*RR* = 3.25, effect coefficient = 0.78, *95% CI*: 0.46–1.10) (Figure 9).

**Table 2:** Age, period, and birthcohort effect coefficients of reported incidence of chronic hepatitis B among residents aged 20–84 years in Chaoyang District.

**Factors**	**Overall**					**Male**					**Female**				
	**Coefficient of influence (** ***95%CI*** **)**	***SE***	***Z***	***P***	***RR***	**Coefficient of influence (** ***95%CI*** **)**	***SE***	***Z***	***P***	***RR***	**Coefficient of influence (** ***95%CI*** **)**	***SE***	***Z***	***P***	***RR***
**Age group (year)**															
20–24	0.121 (−0.068 to 0.311)	0.097	1.26	0.209	1.000	−0.200 (−0.452 to 0.052)	0.129	−1.56	0.120	1.000	0.592 (0.296 to 0.888)	0.151	3.92	0.000	1.000
25–29	0.425 (0.278 to 0.573)	0.075	5.65	0.000	1.355	0.355 (0.167 to 0.542)	0.096	3.71	0.000	1.742	0.565 (0.321 to 0.808)	0.124	4.54	0.000	0.973
30–34	0.132 (−0.011 to 0.275)	0.073	1.81	0.071	1.011	0.169 (−0.009 to 0.347)	0.091	1.86	0.063	1.447	0.090 (−0.152 to 0.332)	0.124	0.73	0.467	0.605
35–39	−0.149 (−0.299 to 0.002)	0.077	−1.93	0.053	0.763	−0.053 (−0.237 to 0.132)	0.094	−0.56	0.577	1.159	−0.294 (−0.559 to -0.029)	0.135	−2.18	0.030	0.412
40–44	−0.401 (−0.571 to -0.230)	0.087	−4.61	0.000	0.593	−0.213 (−0.418 to -0.009)	0.104	−2.05	0.041	0.987	−0.792 (−1.120 to -0.464)	0.167	−4.74	0.000	0.251
45–49	−0.465 (−0.646 to -0.284)	0.092	−5.03	0.000	0.556	−0.370 (−0.593 to -0.146)	0.114	−3.24	0.001	0.844	−0.604 (−0.925 to -0.283)	0.164	−3.69	0.000	0.302
50–54	−0.620 (−0.82 to -0.420)	0.102	−6.08	0.000	0.476	−0.474 (−0.723 to -0.224)	0.127	−3.73	0.000	0.761	−0.846 (−1.197 to -0.495)	0.179	−4.72	0.000	0.237
55–59	−0.187 (−0.398 to 0.024)	0.108	−1.73	0.083	0.735	−0.077 (−0.345 to 0.191)	0.137	−0.56	0.573	1.131	−0.326 (−0.688 to 0.037)	0.185	−1.76	0.078	0.399
60–64	0.076 (−0.163 to 0.314)	0.122	0.62	0.534	0.955	0.098 (−0.212 to 0.409)	0.159	0.62	0.535	1.348	0.128 (−0.258 to 0.514)	0.197	0.65	0.516	0.629
65–69	0.231 (−0.008 to 0.471)	0.122	1.89	0.059	1.116	0.268 (−0.049 to 0.586)	0.162	1.66	0.098	1.598	0.286 (−0.090 to 0.663)	0.192	1.49	0.136	0.736
70–74	0.195 (−0.074 to 0.463)	0.137	1.42	0.155	1.076	0.333 (−0.022 to 0.688)	0.181	1.84	0.066	1.705	0.110 (−0.318 to 0.539)	0.218	0.50	0.614	0.618
75–79	0.354 (0.063 to 0.644)	0.148	2.39	0.017	1.261	0.534 (0.153 to 0.915)	0.194	2.75	0.006	2.084	0.114 (−0.381 to 0.609)	0.253	0.45	0.652	0.620
80–84	0.288 (−0.137 to 0.713)	0.217	1.33	0.185	1.181	−0.372 (−1.122 to 0.378)	0.383	−0.97	0.332	0.842	0.977 (0.436 to 1.517)	0.276	3.54	0.000	1.469
**Period (year)**															
2005	1.172 (1.100 to 1.245)	0.037	31.56	0.000	1.000	0.155 (0.052 to 0.258)	0.047	25.22	0.000	1.000	−0.828 (−1.008 to -0.648)	0.064	18.04	0.000	1.000
2010	0.180 (0.098 to 0.263)	0.042	4.28	0.000	0.371	−0.738 (−0.862 to -0.613)	0.053	2.94	0.003	0.359	−0.56 (−0.739 to -0.382)	0.071	3.33	0.001	0.399
2015	−0.768 (−0.870 to -0.666)	0.052	−14.71	0.000	0.144	−0.598 (−0.729 to -0.466)	0.064	−11.57	0.000	0.147	−0.397 (−1.228 to 0.435)	0.092	−9.03	0.000	0.138
2020	−0.584 (−0.689 to -0.479)	0.054	−10.91	0.000	0.173	0.791 (−0.138 to 1.720)	0.067	−8.91	0.000	0.169	0.21 (−0.251 to 0.671)	0.091	−6.15	0.000	0.180
**Birth cohort (year)**															
1921–1925	0.136 (−0.458 to 0.730)	0.303	0.45	0.654	1.000	−0.323 (−0.743 to 0.097)	0.474	1.67	0.095	1.000	−0.352 (−0.802 to 0.098)	0.424	−0.94	0.350	1.000
1926–1930	0.178 (−0.173 to 0.529)	0.179	1.00	0.319	1.043	−0.051 (−0.404 to 0.302)	0.236	1.12	0.262	0.591	−0.206 (−0.642 to 0.230)	0.305	−0.33	0.739	1.343
1931–1935	−0.095 (−0.398 to 0.209)	0.155	−0.61	0.541	0.794	−0.371 (−0.747 to 0.005)	0.214	−1.51	0.131	0.328	0.321 (−0.059 to 0.702)	0.235	0.89	0.372	1.835
1936–1940	−0.048 (−0.307 to 0.210)	0.132	−0.37	0.715	0.832	−0.181 (−0.531 to 0.168)	0.180	−0.28	0.777	0.431	0.316 (−0.063 to 0.694)	0.205	−0.26	0.794	1.410
1941–1945	−0.381 (−0.662 to -0.100)	0.143	−2.66	0.008	0.596	0.197 (−0.115 to 0.51)	0.192	−1.94	0.053	0.313	0.380 (0.000 to 0.759)	0.229	−1.53	0.125	1.046
1946–1950	−0.194 (−0.458 to 0.070)	0.135	−1.44	0.150	0.719	0.385 (0.091 to 0.679)	0.178	−1.02	0.310	0.378	0.782 (0.459 to 1.105)	0.223	−0.93	0.355	1.210
1951–1955	0.226 (−0.007 to 0.460)	0.119	1.90	0.058	1.094	0.536 (0.264 to 0.807)	0.160	1.24	0.216	0.552	0.736 (0.455 to 1.017)	0.194	1.65	0.098	2.050
1956–1960	0.348 (0.125 to 0.571)	0.114	3.06	0.002	1.237	0.637 (0.389 to 0.886)	0.150	2.56	0.010	0.666	0.617 (0.366 to 0.869)	0.193	1.64	0.102	2.039
1961–1965	0.497 (0.289 to 0.706)	0.106	4.67	0.000	1.435	0.453 (0.220 to 0.687)	0.139	3.87	0.000	0.775	−0.025 (−0.271 to 0.221)	0.194	1.96	0.050	2.174
1966–1970	0.677 (0.491 to 0.864)	0.095	7.11	0.000	1.719	0.340 (0.121 to 0.560)	0.127	5.02	0.000	0.858	−0.208 (−0.501 to 0.094)	0.165	4.75	0.000	3.251
1971–1975	0.538 (0.368 to 0.708)	0.087	6.20	0.000	1.495	0.034 (−0.189 to 0.257)	0.119	3.81	0.000	0.714	−1.282 (−1.830 to -0.734)	0.143	5.14	0.000	3.105
1976–1980	0.436 (0.279 to 0.592)	0.080	5.47	0.000	1.350	−0.172 (−0.448 to 0.104)	0.112	3.03	0.002	0.637	−0.737 (−1.848 to 0.373)	0.128	4.81	0.000	2.757
1981–1985	0.000 (−0.156 to 0.157)	0.080	0.00	0.996	0.873	−0.804 (−1.233 to -0.376)	0.114	0.30	0.766	0.469	−9.149 (−9.279 to -9.019)	0.126	−0.20	0.841	1.450
1986–1990	−0.208 (−0.405 to -0.012)	0.100	−2.08	0.038	0.709	−1.736 (−3.581 to 0.110)	0.141	−1.22	0.223	0.382	1.000 (−3.581 to 0.110)	0.154	−1.35	0.178	1.208
1991–1995	−1.019 (−1.350 to -0.688)	0.169	−6.03	0.000	0.315	−8.566 (−8.713 to -8.419)	0.219	−3.68	0.000	0.203	−8.566 (−8.713 to -8.419)	0.279	−4.59	0.000	0.413
1996–2000	−1.092 (−2.031 to -0.154)	0.479	−2.28	0.022	0.293	1.000 (−2.031 to -0.154)	0.942	−1.84	0.065	0.080	1.000 (−2.031 to -0.154)	0.566	−1.30	0.193	0.711
**Intercept**	−8.780 (−8.869 to -8.692)	0.045	−194.48	0.000	-	−8.780 (−8.869 to -8.692)	0.075	−114.53	0.000	-	−8.780 (−8.869 to -8.692)	0.066	−138.04	0.000	-

## 4 Discussion

During the period 2005–2022, the reported incidence rate of chronic hepatitis B in the resident population of Chaoyang District, Beijing, generally exhibited a decreasing trend, peaking in 2006, likely attributed to the implementation of the “Norms for the Management of Reporting of Information on Infectious Diseases” in 2006, which led to the reporting of previously accumulated unreported cases. Since 2013, the reported incidence rate has stabilized, fluctuating between 7.22 and 10.46 per 100,000. Regarding gender distribution, the male-to-female ratio of reported chronic hepatitis B incidence in Chaoyang District from 2005 to 2022 ranged from 1.24 to 2.11. Biological differences between males and females in terms of immune response to the HBV virus (13), as well as differences in lifestyle or behaviors such as smoking and drinking among males, may contribute to increased liver burden (14, 15). In terms of age distribution, reported cases of chronic hepatitis B were predominantly concentrated in the 25–44 age group. This group represents an immunization gap population as the hepatitis B vaccine was not included in the immunization program when they were born, posing a higher risk of HBV infection (3). Additionally, high social activity during this age range and mandatory medical examinations for employment may lead to higher detection rates of hepatitis B. Moreover, the intense labor and poor lifestyle habits prevalent in this age group contribute to liver disease symptoms and increased medical treatment-seeking behaviors, thereby contributing to higher reported incidence rates (15).

The natural history of hepatitis B differs significantly between acute and chronic forms. Chronic hepatitis B resembles traditional chronic non-communicable diseases in terms of disease onset, progression, and management. Therefore, this study focused on chronic hepatitis B incidence data, analyzed using the age-period-cohort model. Trend analysis of age-period-birth cohort data showed that the reported incidence rate of chronic hepatitis B in the entire 20–84-year-old population of Chaoyang District declined continuously from 2005 to 2015, with a slight rebound in 2020 (Figure 4). This trend was consistent with the hepatitis B surface antigen positivity rate observed in serologic surveys conducted in Chaoyang District (2.61% in 2010, 2.50% in 2015, and 2.52% in 2020) (3). The period effect coefficient shifted from positive to negative values, indicating a gradual decrease in the contribution of period factors to hepatitis B incidence from 2015 to 2020. Since 1992, China has implemented comprehensive hepatitis B prevention and control measures, including vaccination, enhanced blood management, and standardized diagnosis and treatment (1). Hepatitis B vaccination has been particularly pivotal, with the introduction of free vaccination in 2002 and supplementary vaccination campaigns for individuals born between 1994 and 2001 from 2009 to 2011.

The cohort effect of reported chronic hepatitis B incidence in the entire population of Chaoyang District initially increased and then decreased across birth cohorts, reaching its peak in the 1966–1970 cohort (effect coefficient = 0.68, *95% CI*: 0.49–0.86) (Table 2). The 1962–1971 birth cohort represents the second baby boom since the founding of the country (16), a period when universal hepatitis B vaccination had not yet been implemented and hepatitis B prevalence was high. Vertical transmission of hepatitis B from mother to child and horizontal transmission during childhood led to a significant number of early HBV infections in children, contributing to the peak cohort effect observed in the 1966–1970 birth cohort.

With reference to the 20–24 year age group, the 2005 period, and the 1921–1925 birth cohorts, the highest effect of chronic hepatitis B incidence was observed in the 75–79 year age group for males (*RR* = 2.08, effect coefficient = 0.53, *95% CI*: 0.15–0.91). Similarly, the highest effect was found in the female chronic hepatitis B incidence in the 1966–1970 birth cohort (*RR* = 3.25, effect coefficient = 0.78, *95% CI*: 0.46–1.10). In older males, poor physical function and long-term chronic HBV infection increase the risk of morbidity. Socially active lifestyles among men, coupled with high labor intensity and poor lifestyle habits such as smoking and alcohol consumption (17), contribute to the manifestation of liver disease symptoms (18). Increased healthcare-seeking behaviors further contribute to the higher reported incidence of chronic hepatitis B in males. Women, on average, live longer than men, which may delay the peak age effect in females compared to males. In this study, the selected population aged 20–84 years likely captured the peak age effect in females beyond 84 years. The female population born in the 1966–1970 cohort faced higher risks of chronic HBV carriage due to the absence of universal hepatitis B vaccination during their birth period. Additionally, historical factors such as shared needles, single plasma collection campaigns, and a pandemic of hepatitis B from 1970 to 1992 contributed to a higher prevalence of chronic HBV infection among women in this cohort (19). During their childbearing years (ages 25–55), these women were socially active, leading to increased clinic visits and subsequent diagnoses of chronic hepatitis B, thereby increasing reported morbidity rates.

A comparison between the serological survey data of hepatitis B and the reported data on chronic hepatitis B reveals a significant disparity of an order of magnitude. Specifically, the positive rate of HBsAg in the whole population of Chaoyang District was 2.61%, 2.50%, and 2.52% in 2010, 2015, and 2020, respectively (16), while the reported incidence rate of chronic hepatitis B was 16.40/100,000 from 2005 to 2022. After the introduction of neonatal hepatitis B vaccine into the immunization program, the reported incidence of hepatitis B among children under 15 years old has decreased to zero. Nevertheless, in the immunization gap population born prior to the inclusion of hepatitis B vaccine, the burden of hepatitis B carriers remains substantial. At present, it is estimated that 79.74 million people in China are HBsAg positive. However, the diagnosis rate of chronic hepatitis B is only 24% (about 19.44 million people), and the treatment rate is 15% (about 2.92 million people) (20). Chronic hepatitis B cases that remain undiagnosed and untreated are at a high risk of progressing to cirrhosis and liver cancer cases, which poses an obstacle to the achievement of the goal of eliminating viral hepatitis. In the next step, our strategy will focus on early detection and early treatment measures to strengthen the monitoring of new chronic hepatitis B cases, expand the scope of hepatitis B screening, improve the diagnosis rate (90%) and treatment rate (80%) of chronic hepatitis B as much as possible. Prompt intervention for individual cases will be crucial to preventing their transformation to cirrhosis and liver cancer, thereby reducing the long-term disease burden.

This study has several limitations. Firstly, the data were sourced from the National Infectious Disease Reporting System, where reported onset times often coincide with diagnosis times rather than actual disease onset times. This discrepancy may affect the accuracy of temporal analyses. Secondly, the endogenous factor method used in this study, while not relying on a priori assumptions to derive parameter solutions, lacks intuitive interpretation of parameter estimates (21). It provides relative insights into parameter magnitudes and trend changes rather than absolute values. Lastly, the study segmented age, period, and cohort groups with 5-year intervals, limiting the ability to discern short-term fluctuations in age, period, and cohort effects with precision. Future research could benefit from narrower age, period, and cohort groupings to better elucidate trends in chronic hepatitis B incidence.

## 5 Conclusion

Our study revealed a significant decline in the reported incidence of chronic hepatitis B in Chaoyang District from 2005 to 2022, stabilizing after 2013. Besides, we found distinct age, period, and cohort effects on the incidence, with higher risks observed in males aged 25–29 and 75–79, and females born in 1966–1970. These findings highlight the importance of targeted surveillance, expanded screening, and improved diagnosis and treatment rates to reduce the long-term chronic hepatitis B burden.

## Data Availability

The raw data supporting the conclusions of this article will be made available by the authors, without undue reservation.
